# Effects of defined voluntary running distances coupled with high‐fat diet consumption on the skeletal muscle transcriptome of male mice

**DOI:** 10.14814/phy2.70170

**Published:** 2025-01-16

**Authors:** Afrina Brishti, Sarah J. Johnson, Daniel G. Palmer, Md Obayed Raihan, Lin Yan, Shanon L. Casperson

**Affiliations:** ^1^ United States Department of Agriculture, Agricultural Research Service Grand Forks Human Nutrition Research Center Grand Forks North Dakota USA; ^2^ Department of Pharmaceutical Sciences, College of Health Sciences and Pharmacy Chicago State University Chicago Illinois USA; ^3^ Present address: Department of Biomedical Sciences, School of Medicine and Health Sciences University of North Dakota Grand Forks North Dakota USA

**Keywords:** high‐fat diet, skeletal muscle, transcriptome, voluntary exercise

## Abstract

Exercise counters many adverse health effects of consuming a high‐fat diet (HFD). However, complex molecular changes that occur in skeletal muscle in response to exercising while consuming a HFD are not yet known. We investigated the interplay between diverse exercise regimes and HFD consumption on the adaptation of skeletal muscle transcriptome. C57BL/6 male mice were randomized into five groups—one sedentary control group and four exercise groups. The exercise groups consisted of an unrestricted running group (8.3 km/day) and three groups that were restricted to 75%, 50%, or 25% of unrestricted running (6.3, 4.2, and 2.1 km/day, respectively). Total RNA was extracted from frozen gastrocnemius muscle for transcriptome analyses. DEG counts were 1347, 1823, 1103, and 1107 and there were 107, 169, 67, and 89 unique genes present in the HFD‐25%, HFD‐50%, HFD‐75%, and HFD‐U, respectively. Comparing exercise groups, we found that exercising at 50% resulted in the most differentially expressed transcripts with the MAPK and PPAR signaling pathways enriched in down‐ and up‐regulated genes, respectively. These results demonstrate that running distance impacts the adaptation of the skeletal muscle transcriptome to exercise and suggest that middle‐distance running may provide the greatest protection against high‐fat diet‐induced stress coupled with exercise.

## Graphical abstract

The findings from this study provides a comprehensive descriptions of skeletal muscle transcriptome that suggest a middle‐distance running exercise resulted in the most significantly altered differentially expressed genes (DEG). The pathways where these DEG are primarily involved are important for regulating stress responses. Therefore, mice assigned to the HFD‐50% may have been better able to handle the impact of diet‐induced stress and may be more efficient at compensating the detrimental effects of HFD consumption.

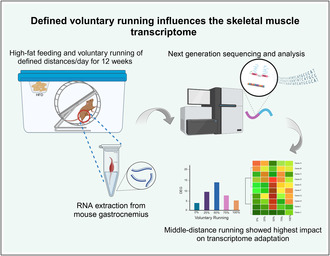

## 1 INTRODUCTION

A significant percentage of Americans regularly ingest a high‐fat diet (a hallmark of the Western Diet) which can lead to innumerable health issues (Campodonico‐Burnett et al., 2020; Rakhra et al., 2020; Shan et al., 2019; Simoes et al., 2020; Woodie et al., 2020). Regular exercise has been shown to reverse many of the negative effects of ingesting a high‐fat diet (Kawanishi et al., 2015; Kim et al., 2016; Lambert et al., 2018; Liu et al., 2015; Touati et al., 2011). Several studies have investigated the interactions of a high‐fat diet and exercise and how this interaction relates to gene expression at the transcriptome level. Xiao et al. (2017) found that exercise capacity is decreased by 71% after only 3 days of consuming a high‐fat diet. Genes involved with immune response were significantly enriched in brain, spleen, and lung tissue while genes related to circulatory system processes and neurotransmission pathways were downregulated in the lung and brain, respectively (Xiao et al., 2017). Messaoudi et al. (2017) conducted a study where rhesus macaques were fed a high‐fat diet for 6 months then fed an energy‐restricted diet for 4 months. They found that while energy restriction resulted in significant weight loss and a decrease in insulin resistance, the dysregulation of genes in skeletal muscle caused by ingestion of a high‐fat diet did not return to healthier levels without exercise. This dysregulation of gene expression included sustained activation of the transforming growth factor beta gene network and enrichment in extracellular matrix remodeling pathways as well as downregulation of genes associated with nutritional processes and muscle structure development (Messaoudi et al., 2017). Pérez‐Schindler et al. (2017) found that pathways involved with remodeling of the extracellular matrix and processes involved with the cellular response to an insulin stimulus were significantly enriched in the skeletal muscle of mice fed a high‐fat diet for 10 weeks after a single bout of maximal exercise. However, gene differentiation in skeletal muscle and the AMPK and PPAR pathways were significantly enriched in control mice in response to a single bout of maximal exercise (Pérez‐Schindler et al., 2017).

The aforementioned studies demonstrate that a high‐fat diet quickly alters the tissue transcriptome resulting in major negative impacts to health. Without exercise, those effects persist even if the diet is changed to be more healthful (Messaoudi et al., 2017; Pérez‐Schindler et al., 2017; Xiao et al., 2017). Ingestion of a high‐fat diet also alters how the body responds to exercise at the transcriptome level. However, when studies involve forced exercise (like acute maximal exercise) a stress response is created that alters the expression of a myriad of molecular pathways (Conner et al., 2014; de Nadal et al., 2011; Hayes et al., 2008; Knab et al., 2009). By using voluntary rather than forced exercise the effects of a stress response are removed and the interplay between ingesting a high‐fat diet and regular exercise can be more clearly elucidated. Therefore, the primary objective of this study was to investigate how daily voluntary aerobic exercise when coupled with ingestion of a high‐fat diet alters the skeletal muscle transcriptome. Of interest is the potential for a dose–response relationship between defined amounts of exercise and ingestion of a high‐fat diet.

We previously reported a dose–response relationship between daily voluntary running of defined distances and reductions in adiposity and its associated inflammation and improvements in insulin sensitivity in mice fed a high‐fat diet (Yan et al., 2017). Specifically, running 8.3 and 6 km/day for 12 weeks resulted in significant loss of fat mass while running 4.2 and 2.1 km/day did not when compared to sedentary mice fed a high‐fat diet. But running only 2.1 km/day was enough to significantly lower the plasma concentration of monocyte chemotactic protein‐1 (MCP‐1) to that observed in sedentary mice fed a AIN93G diet. These findings demonstrate that daily voluntary running of defined distances differentially offsets the deleterious effects of ingesting HFD. To gain a deeper understanding of these differential effects, we analyzed the transcriptome (RNA‐seq) of the gastrocnemius muscle collected in this study. The transcriptome of the gastrocnemius muscle was analyzed because of its significant engagement in exercise movements and its stronger adaptive response to persistent exercise (Schoenfeld et al., 2020).

## 2 MATERIALS AND METHODS

### 2.1 Animals and diets

Three‐ to four‐week‐old male C57BL/6 mice (Harlan, Madison, WI, USA) were housed in a pathogen‐free room at 22 ± 1°C on a 12:12 h light–dark cycle. The parent study was limited to males due to differing responses of female mice to exercise and high‐fat‐diet (Glenmark et al., 2004; Holcomb et al., 2022; Oydanich et al., 2019; Toniolo et al., 2024). Mice were provided with a standard AIN93G diet during acclimation period and a high‐fat diet (HFD) providing 16% and 45% of energy from soybean oil during experimental period. Both pelleted diets were prepared by our in‐house animal diet kitchen staff following the AIN93G formulation (Reeves et al., 1993). The exact composition of the diets is presented in Table 1 and the pelleted diets were stored at −20°C (Yan et al., 2017). Fresh pellets and deionized water were provided to mice every other day. All procedures with experimental animals were approved by the Department of Agriculture, Agricultural Research Service, Grand Forks Human Nutrition Research Center Institutional Animal Care and Use Committee (11/12/2015).

**TABLE 1:** Composition of experimental diets.

	High‐fat diet	AIN93G
Ingredients, g/kg		
Corn starch	42.5	397.5
Casein	239.4	200
Dextrin	239.4	132
Sucrose	119.7	100
Soybean oil	239.4	70
Cellulose	59.8	50
Ain93 mineral mix	41.9	35
AIN93 vitamin mix	12	10
L‐Cystine	3.6	3
Choline bitartrate	3	2.5
t‐Butylhydroquinone	0.017	0.014
Total	1000	1000
Energy%		
Protein	20	20
Fat	45	16
Carbohydrate	35	64
Analyzed gross energy, Kcal/g	5.2 ± 0.1	4.3 ± 0.1

### 2.2 Experimental design

**FIGURE 1:**
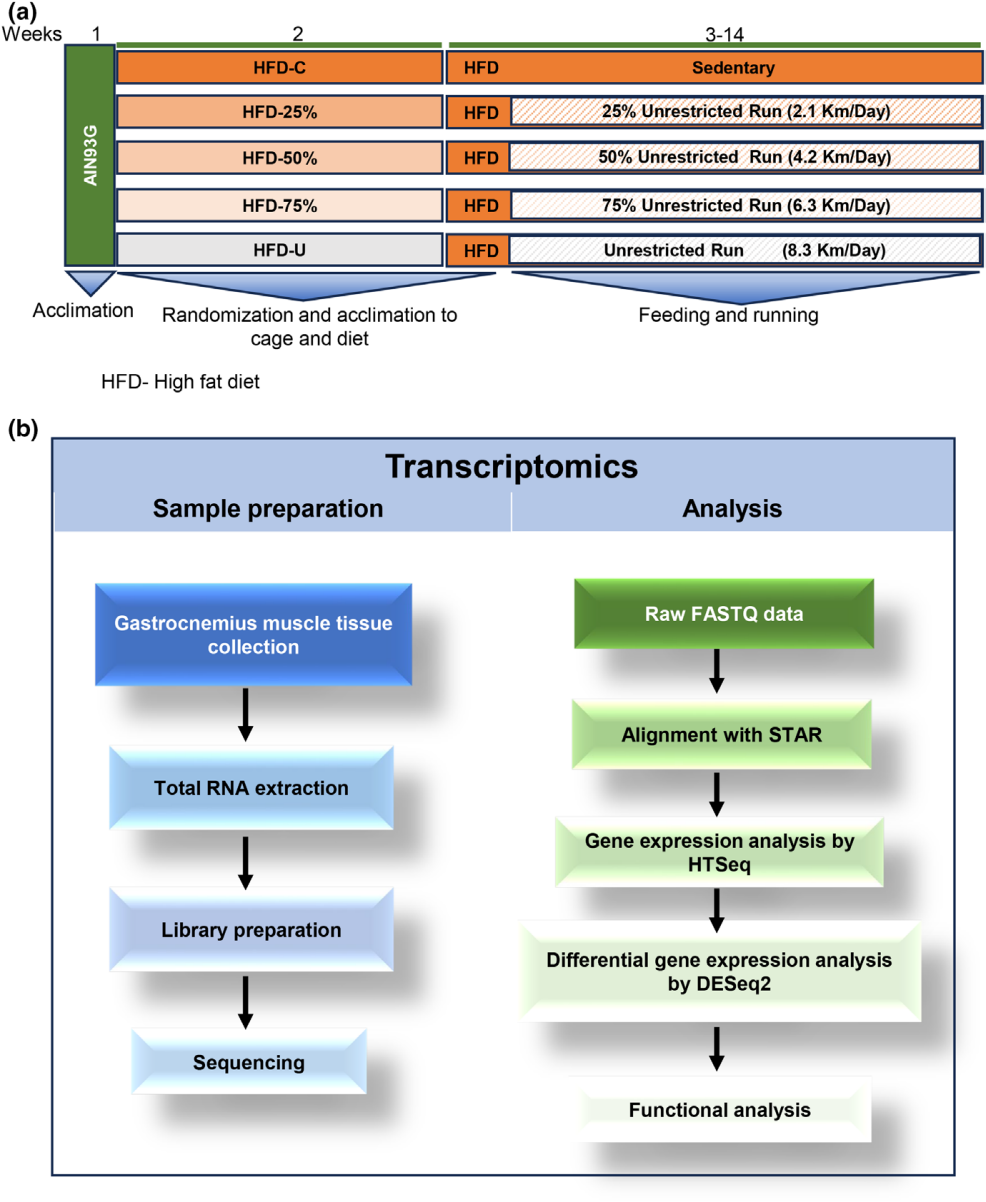
Method outline. (a) Schematic diagram for the experimental modeling of animals. (b) Flowchart of sample preparation (blue) and analytical methods (green) for RNA‐sequencing.

The experimental design has been published (Yan et al., 2017). The Institutional Animal Care and Use Committee of the Grand Forks Human Nutrition Research Center reviewed and approved this study. Briefly, mice were acclimated with the AIN93G diet for 1 week before being randomly assigned to one of five groups—one sedentary control groups and four exercise groups. The sedentary control group and four exercise groups were fed the HFD (Table 1). The four exercise groups had access to an in‐cage running wheel equipped with programmable brakes (Model 86120, Scurry Activity Monitoring Systems, Lafayette Instrument, Lafayette, Ind.). One group had unrestricted access to the running wheel and averaged 8.3 km/day (HFD‐U). The running distance of the HFD‐U was then used to calculate the running distance of the other exercise groups so that running was restricted by 75%, 50%, or 25% with average running distances of 6.3, 4.2, and 2.1 km/day, respectively (Figure 1a). Brakes were released at Zeitgeber time 12 (light off) for restricted runners and applied when they met their assigned daily running distances. After 12 weeks the mice were euthanized by an intraperitoneal injection of a mixture of ketamine (100 mg per kg body weight) and xylazine (10 mg per kg body weight) followed by exsanguination. Gastrocnemius muscles from both legs were collected, flash frozen, and stored at −80°C.

### 2.3 RNAseq analysis

One of the gastrocnemius muscles from each mouse was pulverized in liquid nitrogen and homogenized using a Tissuelyzer LT (Qiagen, Valencia, CA, USA). Total RNA was extracted using the RNeasy Lipid Tissue Mini Kit (Cat#74804, Qiagen, Valencia, CA, USA). RNA quality was determined using the Fragment Analyzer RNA Kit (DNF‐471, Agilent, Santa Clara, CA, USA) on the 5200 Fragment Analyzer System (Agilent, Santa Clara, CA, USA) (Figure 1b). Ten nanograms of each sample with a higher than 6.3 RNA Quality Number (*n* = 6 per group) was sent to Novogene (Sacramento, CA, USA) for sequencing and enrichment analyses. RNA‐seq library preparation, sequencing, and differential gene expression analyses were performed by Novogene (Beijing, China) using Illumina's TruSeq RNA Library Prep Kit and the Illumina HiSeq 4000 platform. The raw sequencing data was processed using Hisat2 v2.0.5 for alignment, featureCounts v1.5.0‐p3 for gene expression quantification, and DESeq2 for differential gene expression analysis. To determine target gene function enrichment analyses, a cutoff of |log2 fold‐change| ≥ 0.0, DESeq2 p‐value ≤0.05, and a Raw count average ≥ 25 was implemented (Figure 1b). And to determine genes for qPCR verification a cut‐off of |log2 fold‐change| ≥ 1.0, DESeq2 FDR‐controlled *p*‐value ≤0.05 was employed. The data generated in this study has been deposited in the NCBI GEO database under accession number GSE275818. Biological pathway and disease information enrichment analyses were performed with Kyoto Encyclopedia of Genes and Genomes (KEGG) database. ClusterProfiler R package was used to test the statistical enrichment of differential expression genes in KEGG pathways.

### 2.4 RT‐qPCR analysis

RNA (1 μg) from the same samples prepared and sent for RNAseq analysis was converted to cDNA using the High‐capacity cDNA reverse transcription kit w/RNase inhibitor (Cat#4374967, Applied Biosystems, Inc., Carlsbad, CA, USA) following the manufacturer's protocols. RT‐qPCR reactions contained the following: 1X SYBR Green Master Mix (Cat#4364346, Applied Biosystem, Inc., Carlsbad, CA, USA), 250 nM of forward and reverse primers (IDT Integrated DNA Technologies, Coralville, IA) (Table 2), and 40 ng cDNA. Real Time PCR was performed on a Quantstudio 12 k flex Real‐Time PCR System (Applied Biosystem, Inc., Carlsbad, CA, USA).

**TABLE 2:** Sequence of primers used for RT‐qPCR with >/= 90% efficiencies.

Gene name	Gene ID		Sequence (5′‐3′)
GAPDH		F	AAT GGT GAA GGT CGG TGT G
		R	GTG GAG TCA TAC TGG AAC ATG TAG
FKBP5	14229	F	GAC ACC AAA GAA AAG CTG ACG
		R	ACT ATC TTC CTG TAC TGA ATC ACG
XDH	22436	F	CTC AAG ACC ACT CCG AAG C
		R	AAT TCC ACC ATC CCA CCA G
PIK3R1	18708	F	CCA CTA CTG TAG CCA ACA ACA
		R	AGT AGA TGC GTC TCG TAC CA
FIBIN	67606	F	GAT GCC TAC TCC AAC TCT GAC
		R	CCT CAT TGA GTC TGC TTT CCT
CAR4	12351	F	CAG AGC ACA GTA TTG ATG GGA
		R	TTC ACC TTG TCT CCT ACC TCA
ALDH1L1	107747	F	TGT ACA ACC GTT TCC TCT TCC
		R	CTG TCT CCT TTT TCT GAA TGC C
ALDH9A1	56752	F	AGT AAG AAA AGT GGC CTG GAG
		R	TTC CCG TTG TTG ATG GTC TC
SESN1	140742	F	CAC CAG CAA GAG ATG TGT CT
		R	CGA TTC ACC AGA GAG TAA CCA T
FOX01	56458	F	GTC AAG ACT ACA ACA CAC AGC
		R	AAA ACT ATA AGG AGG GGT GAA GG
PHYHD1	227696	F	GCA GAG GAT CGG TGA GAT TG
		R	CGC TGC TCA AGA AGT AGT CTG
KLF10	21847	F	CCA ACC ATG CTC AAC TTC G
		R	TCA AAG TCA CTC TGC TCA GC
LARS2	102436	F	GGA AGG ATG TGG AGA AGT GG
		R	GGA ACA TGG AGA GCA AGT AGA

### 2.5 Statistical analysis of phenotype variables

**FIGURE 2:**
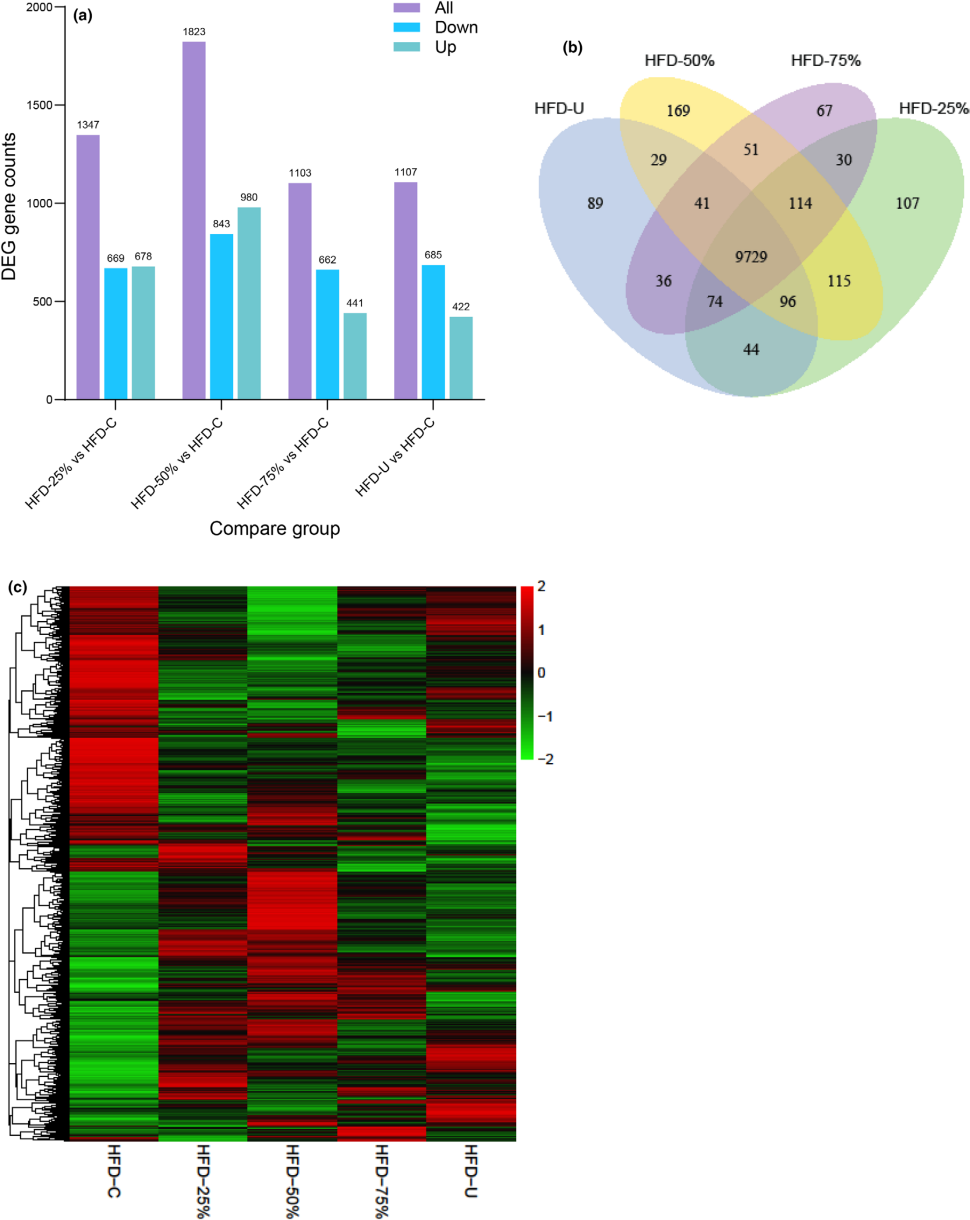
Differentially expressed gene (DEG) counts heatmap. (a) DEG plotted for all, upregulated, and downregulated genes in HFD‐25%, HFD‐50%, HFD‐75%, and HFD‐U compared to HFD‐C. Numbers indicated on the top of each bar represents the gene counts with the threshold level DESeq2 *p*‐value ≤0.05. (b) Venn diagram with four overlapping circles, each represents a different pairwise comparison. Here green represents HFD‐C versus HFD+ 25% exercise group, Yellow HFD‐C versus HFD+ 50% exercise group, purple HFD‐C versus HFD+ 75% exercise group, and blue HFD‐C versus the HFD+ 100% exercise group. The numbers within each circle and overlap represent the number of differentially expressed genes (DEGs) unique to that comparison or shared between multiple comparisons, respectively. (c) Heatmap representing the expression level of 4048 differentially expressed genes among all five experimental groups. We used the mainstream hierarchical clustering to cluster the FPKM values of genes and homogenized the row (Z‐score). The color in each grid reflects not the gene expression value but the value obtained after homogenizing the expression data rows (generally between −2 and 2). The colors in the heat map can only be compared horizontally (the expression of the same gene in different samples) and not vertically (the same sample). Red indicates upregulated genes and green indicates downregulated genes.

Statistical analysis was performed on all normalized and log2‐transformed data to determine differentially expressed genes by R package DESeq2. The resulting *p*‐values were adjusted using the Benjamini and Hochberg's approach for controlling the false discovery rate. Genes with an *p*‐value <=0.05 found by DESeq2 were assigned as differentially expressed in Figure 2a. Pheatmap package in R was utilized to perform the hierarchical cluster analysis (Tang et al., 2023). Dose response omics analysis was carried out in R by using DRomics, a turnkey tool and supports the use of the dose–response framework for omics data (Larras et al., 2018). The RT‐qPCR results were analyzed using one‐way ANOVA and Tukey's post hoc in GraphPad Prism (San Diego, CA, USA). A significance level of *α* = 0.05 was used. Data are reported as the mean ± standard deviation.

## 3 RESULTS

In comparing the HFD‐C with all the exercise groups combined we found that 1094 genes were differentially expressed (DEG). However, when comparing the HFD‐C with each exercise group separately we found that the DEG counts varied in response to the different running distances. As shown in Figure 2a, the total DEG counts were 1347, 1823, 1103, and 1107 in the HFD‐25% (2.1 km/day), HFD‐50% (4.2 km/day), HFD‐75% (6.3 km/day), and HFD‐U (8.3 km/day), respectively. Of interest were the different patterns observed in the down‐ and up‐regulated DEG counts between the exercise groups. In the HFD‐50% there were more upregulated than downregulated DEG counts which is the reverse of that observed in the HFD‐75% and HFD‐U groups.

Venn diagram in Figure 2b with four overlapping circles represents sets of the number of differentially expressed genes (DEGs) for each HFD exercise group compared to the HFD control group. There were 9729 overlapping genes found in all the exercise groups whereas 107,169,67 and 89 unique genes were present in the HFD‐25%, HFD‐50%, HFD‐75% and HFD‐U groups, respectively.

**FIGURE 3:**
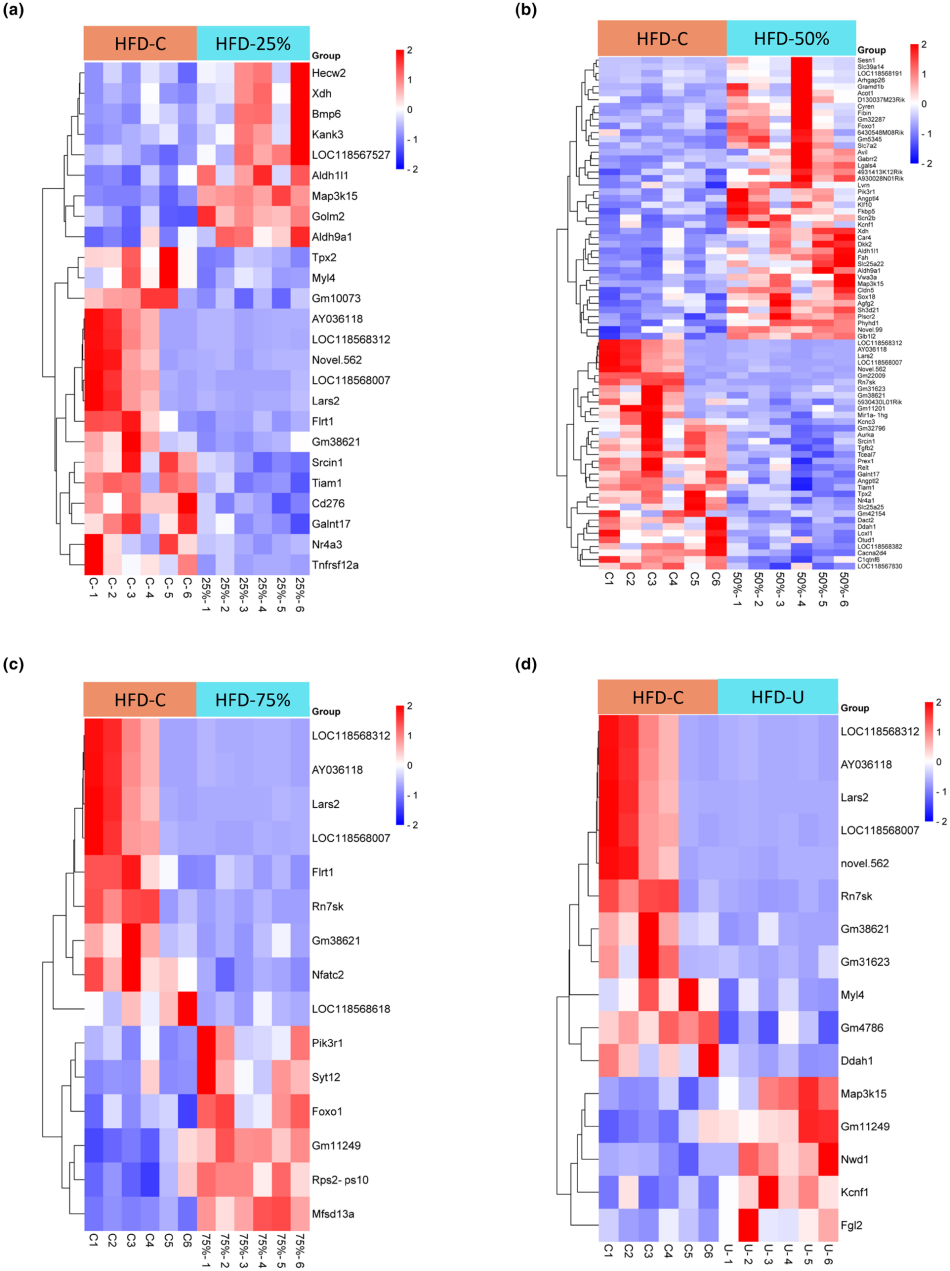
Hierarchical clustering heatmap of differentially expressed genes using pheatmap package. There are 25, 78, 15, and 16 genes identified in HFD‐25%, HFD‐50%, HFD‐75%, and HFD‐U groups, respectively, compared to HFD‐C. DESeq2 FDR‐controlled *p*‐value ≤0.05, |log2FoldChange| ≥ 1.0. Red indicates upregulated genes and blue indicates downregulated genes (*n* = 6 mice per group).

Figure 2c shows the hierarchical clustering heatmap visualization of the DEG pattern for each group. Hierarchical clustering analysis was used to compare genes with similar expression patterns between the HFD‐C and the exercise groups. The most differentially expressed (|log2FoldChange| > =1.0) genes, DESeq2 FDR‐controlled *p*‐value ≤0.05 between groups at each of the running distances are presented in Figure 3. Comparing the HFD‐C and the HFD‐25%, HFD‐50%, HFD‐75%, and HFD‐U groups, there are 25, 78, 15, and 16 genes identified, respectively. Notably, the HFD‐50% had the most differentially expressed genes.

**FIGURE 4:**
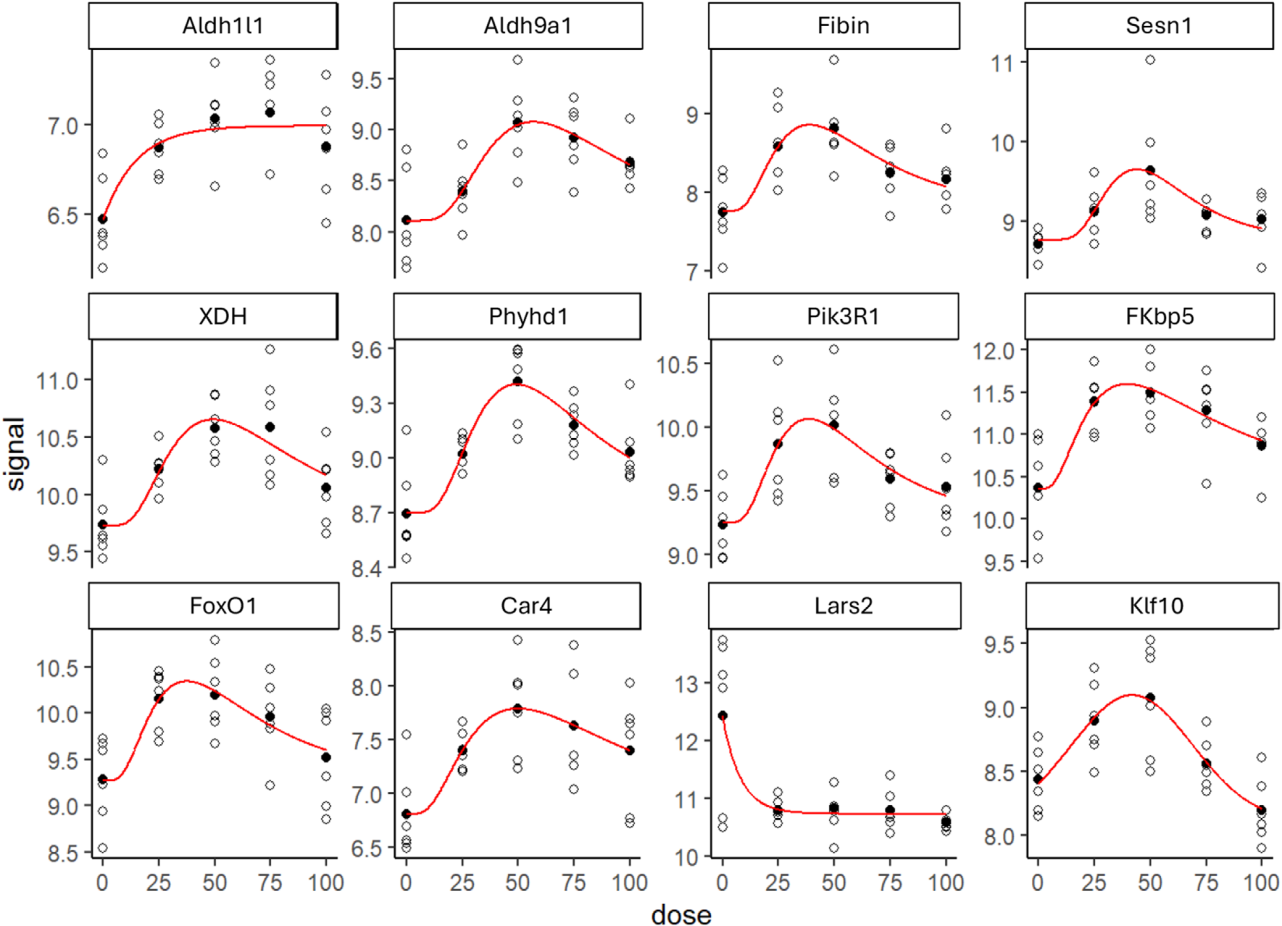
Dose–response framework for omics data from defined running distances. The 12 dose–response curves among the 286 best‐fit models are shown. Daily running distance is presented on the y‐axis as the dose. Distribution of the chosen models was calculated using Hill = 0, linear = 16, exponential = 57, Gauss‐probit = 13, log‐Gauss‐probit = 200 and distribution of the trends (curve shapes) were calculated using bell‐104, decreasing‐39, increasing‐34, and U‐109. Significant dose–response curves were determined from the adjusted *p*‐values.

We used the DRomics package for R to do the dose–response framework to determine whether there was any effect of different running distances on the transcriptome. Using the AICc to select the best‐fit model, 28 dose–response curves out of 314 previously selected were removed because no model could be fitted reliably. Distribution of the chosen models among the 286 fitted dose–response curves were calculated using Hill =0, linear =16, exponential =57, Gauss‐probit =13, and log‐Gauss‐probit =200. Distribution of the trends (curve shapes) among the 286 fitted dose–response curves were calculated using bell‐104, decreasing‐39, increasing‐34, and U‐109. From the 286 genes identified from fitted dose–response curves the Aldh1l1, Aldh9a1, Fibin, Sesn1, XHD, Phyhd1, Pik3R1, FKbp5, Foxo1, Car4, Lars2, and Klf10 were found to be significant. Among these 12 dose–response curves we found that the HFD‐50% had the highest alteration in transcription (Figure 4).

**FIGURE 5:**
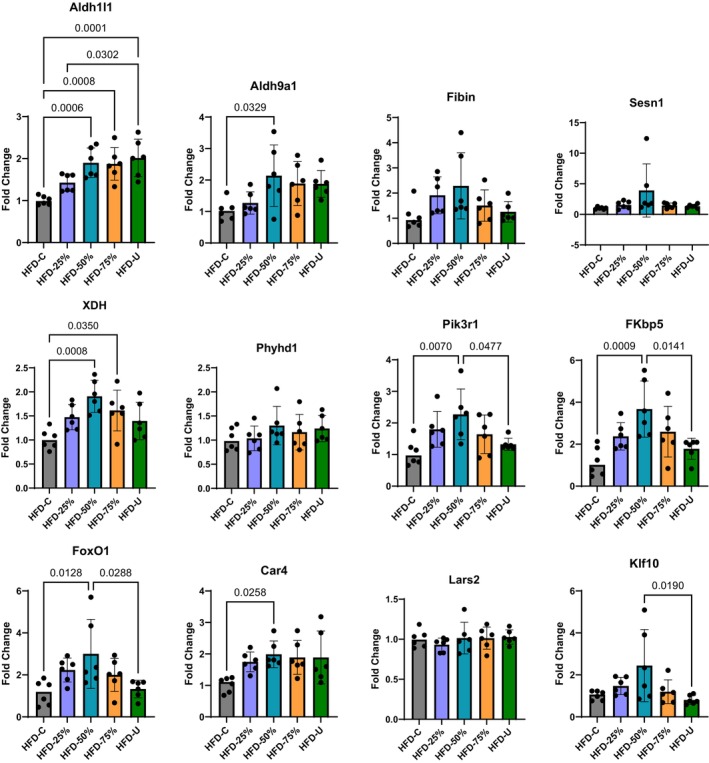
RNA expression patterns from the qPCR analysis. Relative expression level fold change of the 12 genes with significant dose–response curves were calculated relative to GAPDH mRNA level. Significant and adjusted *p*‐values are shown from the One‐way ANOVA, *n* = 6 per group. Data are presented as the mean ± SD.

We used real‐time quantitative PCR (RT‐qPCR) analysis to validate the 12 significantly altered genes between the exercise groups and HFD‐C in our RNA‐seq data. The fold change graphs are shown in Figure 5. The RNA expression level of aldehyde dehydrogenase 1 family member L1 (Aldh1l1) was greater in the HFD‐50% (1.900 ± 0.352; *p* = 0.0006), HFD‐75% (1.874 ± 0.388; *p* = 0.0008), and the HFD‐U (2.015 ± 0.446; *p* = 0.0001) compared to the HFD‐C (1.004 ± 0.104). Whereas the RNA expression level of xanthine dehydrogenase (XHD) was greater in the HFD‐50% (1.908 ± 0.335; *p* = 0.0008) and HFD‐75% (1.613 ± 0.424; *p* = 0.035) compared to the HFD‐C (1.017 ± 0.207). Only the HFD‐50% had greater RNA expression levels of carbonic anhydrase 4 (Car4) (1.988 ± 0.424; *p* = 0.0258) and aldehyde dehydrogenase 9 family member A1 (Aldh9a1) (2.139 ± 0.976; *p* = 0.0329) compared to HFD‐C (1.025 ± 0.238 and 1.039 ± 0.324, respectively). However, the RNA expression level of Phosphoinositide‐3‐kinase regulatory subunit 1 (Pik3r1) was greater in the HFD‐50% (2.273 ± 0.802) compared to both the HFD‐C (1.059 ± 0.409; *p* = 0.007) and HFD‐U (1.325 ± 0.194; *p* = 0.0477) groups. FKBP prolyl isomerase 5 (FKBP5) RNA expression level was also greater in the HFD‐50% (3.672 ± 1.338) compared to both the HFD‐C (1.169 ± 0.689; *p* = 0.0009) and HFD‐U (1.779 ± 0.503; *p* = 0.0141) groups. Forkhead box O1 (FoxO1) RNA expression was greater in HFD‐50% (3.001 ± 1.641) compared to both the HFD‐C (1.141 ± 0.587; *p* = 0.0128) and HFD‐U (1.325 ± 0.416; *p* = 0.0288) groups as well. KLF transcription factor 10 (Klf10) RNA expression levels was greater in the HFD‐50% (2.443 ± 1.719; *p* = 0.019) only when compared to HFD‐U (1.020 ± 0.217). The RNA expression level of Fin bud initiation factor homolog (Fibrin), sestrin 1 (Sesn1), and phytanoyl‐CoA dioxygenase domain containing 1 (Phyhd1) were not significantly different, possibly due to the large variation between samples.

**FIGURE 6:**
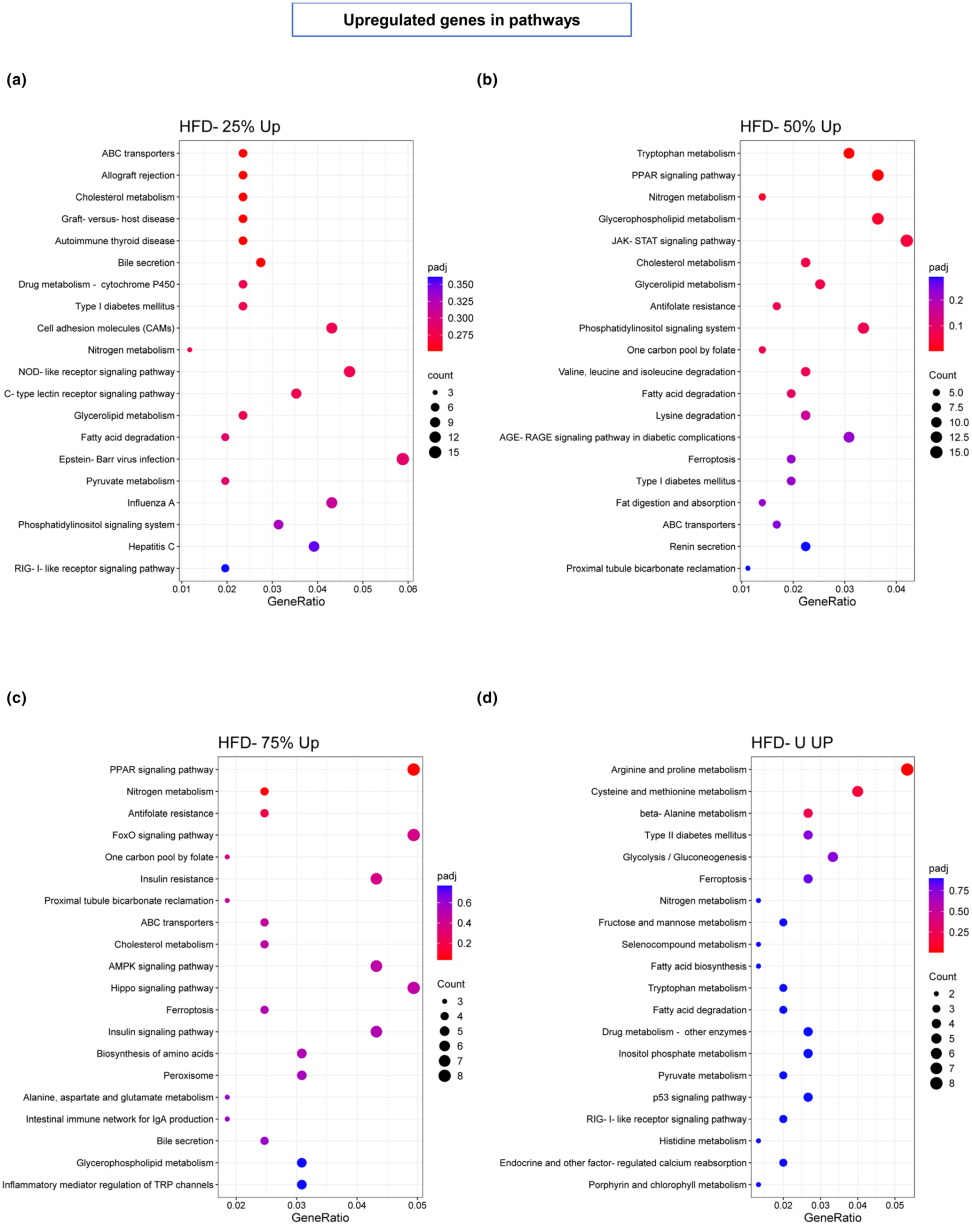
The 20 most up‐regulated pathways and biological functions by the KEGG enrichment analysis of differentially expressed genes. (a) HFD‐25%, (b) HFD‐50%, (c) HFD‐75%, and (d) HFD‐U groups are plotted in the bubble plot where the reference group was HFD‐C; *n* = 6 per group. The color panel represents the adjusted *p*‐value with red indicates greater significance. The different dot sizes represent the number of differentially expressed genes in that pathway.

**FIGURE 7:**
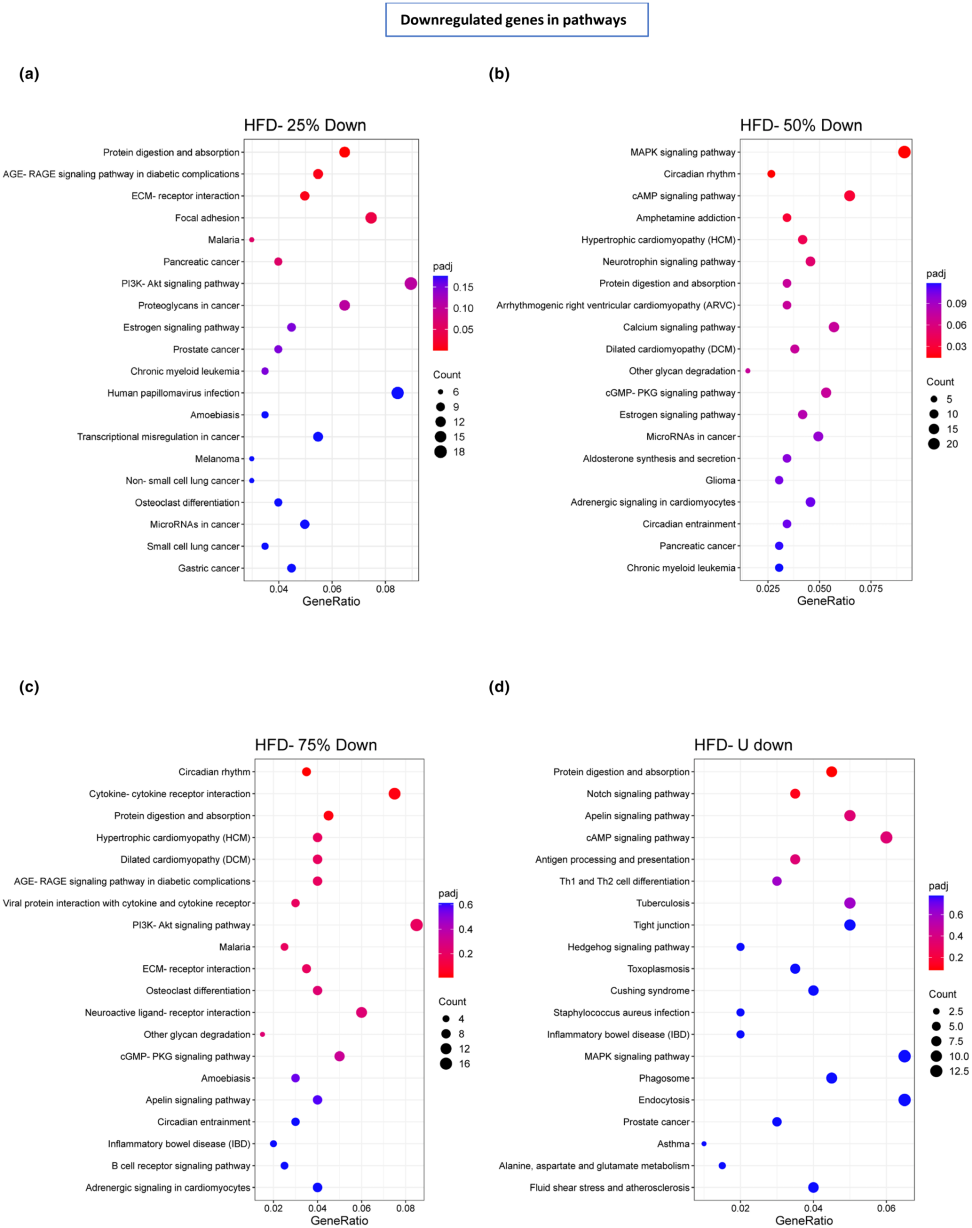
The 20 most downregulated pathways and biological functions by the KEGG enrichment analysis of differentially expressed genes. (a) HFD‐25%, (b) HFD‐50%, (c) HFD‐75%, and (d) HFD‐U groups are plotted in the bubble plot where the reference group was HFD‐C; *n* = 6 per group. The color panel represents the adjusted *p*‐value with red indicates greater significance. The different dot sizes represent the number of differentially expressed genes in that pathway.

To identify the pathways potentially involved in these genetic changes, we performed KEGG analysis of the upregulated (Figure 6) and downregulated (Figure 7) genes separately. In the HFD‐25% the upregulated genes were involved in ABC transporter, cholesterol metabolism, allograft rejection, and nitrogen metabolism (Figure 6a) while the downregulated genes were mostly involved with protein digestion and absorption, AGE‐RAGE signaling, and cholesterol metabolism (Figure 7a). In the HFD‐50% the upregulated genes were involved in tryptophan, cholesterol, glycerolipid, glycerophospholipid, and nitrogen metabolism, PPAR, JAK–STAT, AGE‐RAGE signaling, phosphatidylinositol signaling, antifolate resistance and fatty acid‐lysine‐valine‐leucine and isoleucine degradation (Figure 6b) while the downregulated genes were involved in MAPK and cAMP signaling, circadian rhythm, amphetamine addiction, hypertrophic cardiomyopathy, protein digestion and absorption, estrogen signaling, cGMP‐PKG signaling, and calcium signaling (Figure 7b). In the HFD‐75%, the upregulated genes were involved in nitrogen metabolism, antifolate resistance, PPAR signaling, and FOXO signaling (Figure 6c) while the downregulated genes were involved in protein digestion and absorption, circadian rhythm, and cytokine–cytokine receptor interaction (Figure 7c). In the HFD‐U, the upregulated genes were involved in arginine & proline metabolism, cysteine & methionine metabolism, and beta‐alanine metabolism (Figure 6d) while the downregulated genes are involved mostly with protein digestion and absorption, cAMP signaling, notch and apelin signaling (Figure 7d).

## 4 DISCUSSION

Elucidating how varying amounts of exercise influence gene expression profile of skeletal muscle tissue is critical for understanding how exercise offsets the effects of consuming an obesogenic diet. Overall, our current study finding that running 4.2 km/day (50% of the unrestricted running group) resulted in the highest number of differentially expressed genes, with the PPAR and MAPK signaling pathways enriched in differentially expressed up‐ and down‐regulated genes, respectively, suggesting possible greater protection against high‐fat diet‐induced stress coupled with exercise (Bouviere et al., 2021; Chen et al., 2022). However, these findings do not directly align with our previous findings using the same model (Yan et al., 2017). In our previous work, we found that running 6 km/day (75% of the unrestricted running group) most effectively reduced adiposity while running 2.1 km/day (25% of the unrestricted running group) was not enough to effectively change body composition (Yan et al., 2017). However, running only 2.1 km/day was enough to significantly lower the plasma concentration of monocyte chemotactic protein‐1 (MCP‐1) to that observed in sedentary mice fed a AIN93G diet. Here we investigated how these same amounts of voluntary aerobic exercise influenced the adaptation of the skeletal muscle transcriptome. Our current findings revealed that running 4.2 km/day (50% of the unrestricted running group) may provide the greatest protection against high‐fat diet‐induced stress through the PPAR and MAPK signaling pathways (Bouviere et al., 2021; Chen et al., 2022). The discrepancies between the two study outcomes might be in part due to the fact that skeletal muscle, as the primary tissue directly involved in exercise training, undergoes significantly greater changes in gene expression resulting in more pronounced genetic adaptations compared to adipose tissue (Hargreaves, 2015). Accordingly, the maximum response observed in adipose tissue with 75% exercise training is evident in skeletal muscle even at 50%. Based on earlier findings linking repeated bouts of aerobic exercise with longer lasting changes in skeletal muscle gene expression (Hargreaves, 2015), the results of this study establish for the first time that running distance has a significant impact on the adaptive response of the skeletal muscle transcriptome.

To better understand the adaptive response of the skeletal muscle transcriptome to daily voluntary running of defined distances, we applied DRomics analysis that identified 286 genes with a dose–response curve. These gene expression patterns help explain the influence of running distance to offset consumption of an obesogenic diet. Among the 12 genes validated, only Aldh1l1 showed a steady increase in expression with increasing running distance when compared to sedentary control. Aldh1l1 is a folate metabolism enzyme recently recognized as a source of oxidative stress in skeletal muscle fibers and plays a critical role in skeletal muscle adaptation to exercise (Dungan et al., 2022). Interestingly, increased expression of 6 of the 12 genes was only observed in the HFD‐50% group. Aldh9a1 is a skeletal muscle enriched gene that catalyzes the oxidation of betaine (Etienne et al., 2020) which is a derivative of the amino acid glycine and known as a regulator of skeletal muscle lipid metabolism (Li et al., 2017). Pik3r1 is essential for the insulin‐stimulated increase in glucose uptake and glycogen synthesis in the muscle (Tsay & Wang, 2023), and loss of Pik3r1 signaling in muscle leads to impaired muscle growth and insulin response (Luo et al., 2006). FKBP5 (FK506‐binding protein) is a stress‐responsive gene that plays a role in energy and glucose homeostasis (Balsevich et al., 2017). A recent investigation demonstrated that FKBP5 deletion in the medio basal hypothalamus strongly induces obesity, whereas its overexpression protects against high‐fat diet induced obesity (Bajaj et al., 2022; Häusl et al., 2022). FoxO1 is a transcription factor that is the main target of insulin signaling and has been implicated in lipid and glucose homeostasis (Pan et al., 2017). FoxO1 dysregulation contributes to many metabolic conditions, especially obesity and an accumulating number of investigations have reported an increase in FoxO1 activities in the adaptation of skeletal muscle to exercise (as reviewed by (Sanchez, 2015)). Klf10, also known as TGFβ‐inducible early gene‐1 is a transcription factor, was recently identified as an important regulator of obesity and insulin resistance (Wara et al., 2020) and acts as a downstream effector of exercise to combat nonalcoholic steatohepatitis (Luo et al., 2024). Car4 (Carbonic anhydrase 4) is a class of metabolic enzymes that play a pivotal role in fatty acid biosynthesis and de novo lipogenesis (Supuran, 2022). Most notably, within the skeletal muscle these genes play a major role in glucose and lipid metabolism.

In the HFD‐50%, the most significantly upregulated pathway was tryptophan metabolism which was not seen in the other running groups. Tryptophan plays a key role in both immunological and neuropsychological processes (Gostner Johanna et al., 2019). Of particular interest is the concurrent significant upregulation of the PPAR signaling pathway. This combination of both tryptophan metabolism and the PPAR signaling pathway may point to this exercise regime providing optimal protection against stress‐induced depression (Agudelo et al., 2014). The most significantly downregulated pathway was the MAPK signaling pathway. One role of the MAPK signaling pathway is regulating the release of inflammatory mediators and downregulation reduces the production of proinflammatory cytokines (Cuenda & Rousseau, 2007) and may play a role in reducing injury to the muscle caused by oxidative stress (Li et al., 2024). Limiting the running distance of these mice to 50% of the unrestricted running distance appears to provide protection against stress that was not observed in the other exercise groups. On the contrary, in the HFD‐25% no pathways were upregulated significantly. But we identified the downregulation of protein digestion and absorption, AGE‐RAGE signaling, and extracellular matrix (ECM)‐receptor interaction pathways as being the most impacted when compared to HFD‐C. AGE‐RAGE signaling plays an important role in skeletal muscle homeostasis of mice under metabolic stress and was mostly common to all running exercise groups as expected (Velayoudom‐Cephise et al., 2020). Genes involved in ECM‐receptor interaction are critical for skeletal muscle force generation and remodeling of ECM interaction following exercise modulates cell signaling essential for skeletal muscle adaptation (Mavropalias et al., 2023). For the HFD‐75%, significant upregulated pathways were PPAR signaling and nitrogen metabolism, whereas the circadian rhythm, cytokine‐cytokine receptor interaction and protein digestion and absorption pathways were significantly downregulated. While not significantly altered, FoxO signaling was one of the involved downregulated pathways. FoxO transcription factors known to modulate the expression of genes involved in PPAR signaling which plays a vital role in exercise to offset high‐fat‐feeding related damages in the skeletal muscle (Jin et al., 2023). Additionally, the activation of PPAR signaling has recently been proposed as a mechanism in exercise‐mediated muscle remodeling (Ferraro et al., 2014). For the HFD‐U, prominent upregulated pathways involved arginine & proline metabolism and downregulated pathways were protein digestion and absorption and notch signaling pathway. Notch signaling regulates insulin transport to muscle and activation of notch signals has been reported to lower insulin sensitivity and increase blood glucose levels, whereas blocking the notch signal improved insulin resistance, and decreased fat accumulation in the skeletal muscle of high‐fat feeding mice (Hasan et al., 2020).

This study is not without limitation. While mice remained physically active outside the running wheel to meet their basic physical needs, our research focused solely on the effects of exercise, without considering the combined influence of exercise and activities of daily living. Because female mice respond differently to exercise and high‐fat‐diets, this study was conducted exclusively on male mice which limits its generalizability. Additionally, the scope was limited to analyzing the transcriptome of the gastrocnemius muscle, chosen for its significant engagement in exercise movements and its pronounced adaptive response to sustained exercise. Moreover, we did not investigate the functional consequences of the observed transcriptional changes on the gastrocnemius muscle or overall health.

### 4.1 Perspectives and significance

Understanding the cellular and molecular mechanisms involved in skeletal muscle adaptation to exercise is essential to optimize evidence‐informed training programs for disease prevention. This is the first investigation of the adaptation of the skeletal muscle transcriptome in response to varying amounts of daily voluntary aerobic exercise in the context of HFD. The results from this study provide detailed characterizations of skeletal muscle transcript signatures revealing that middle‐distance running (50% of unrestricted running or 4.2 km/day) may provide the greatest protection against high‐fat diet‐induced stress coupled with exercise. Eating a HFD can cause stress‐related effects within the body, one of them being a state of chronic low‐grade inflammation (Duan et al., 2018; Tan & Norhaizan, 2019). Therefore, mice assigned to the HFD‐50% may have been better able to handle the impact of diet‐induced stress/inflammation. More research is needed to confirm these findings and to determine how this might extent to a human model.

## AUTHOR CONTRIBUTIONS

SLC and SJJ conceived and designed the research presented in this paper; LY conceived and designed the research from which the skeletal muscles were collected; SJJ performed experiments; AB, SJJ and DGP analyzed data; AB, SJJ, MOR and SLC interpreted results of experiments; AB prepared figures; AB and MOR drafted manuscript; all authors edited and revised the manuscript and approved this final version.

## FUNDING INFORMATION

US Department of Agriculture, Agricultural Research Service #3062‐10700‐001‐000D (to SLC) and 3062‐10700‐002‐000D (to LY).

## CONFLICT OF INTEREST STATEMENT

The authors have nothing to disclose.

## DISCLAIMERS

The findings and conclusions in this publication are those of the author(s) and should not be construed to represent any official USDA or U.S. Government determination or policy.

## Data Availability

All data will be provided upon request. The raw transcriptome data has been deposited in the NCBI GEO database under accession number GSE275818.
